# Can Unconventional Immunomodulatory Agents Help Alleviate COVID-19 Symptoms and Severity?

**DOI:** 10.1128/mSphere.00288-20

**Published:** 2020-05-13

**Authors:** Stephen W. Mamber, Steven Krakowka, Jeffrey Osborn, Lloyd Saberski, Ryan G. Rhodes, Albert E. Dahlberg, Sunthorn Pond-Tor, Kara Fitzgerald, Neal Wright, Sarah Beseme, John McMichael

**Affiliations:** a The Institute for Therapeutic Discovery, Delanson, New York, USA; b The Ohio State University, Columbus, Ohio, USA; c The University of Kentucky, Lexington, Kentucky, USA; d Yale University, New Haven, Connecticut, USA; e The University of North Carolina—Wilmington, Wilmington, North Carolina, USA; f Brown University, Providence, Rhode Island, USA; g Sandy Hook Clinic, Sandy Hook, Connecticut, USA; h CMC Biosciences, Beverly, Massachusetts, USA; i Beech Tree Labs, Providence, Rhode Island, USA; National Institute of Allergy and Infectious Diseases

**Keywords:** COVID-19, SARS-CoV-2, immunomodulatory agents, interferon alpha, phytocannabinoids, thimerosal

## Abstract

Severe acute respiratory syndrome coronavirus 2 (SARS coronavirus 2, or SARS-CoV-2) is the cause of the respiratory infection known as COVID-19. From an immunopathological standpoint, coronaviruses such as SARS-CoV-2 induce increased levels of a variety of T-helper 1 (Th1) and inflammatory cytokines and chemokines, including interleukin-1 (IL-1), IL-6, CCL2 protein, and CXCL10 protein. In the absence of proven antiviral agents or an effective vaccine, substances with immunomodulatory activity may be able to inhibit inflammatory and Th1 cytokines and/or yield an anti-inflammatory and/or Th2 immune response to counteract COVID-19 symptoms and severity. This report briefly describes the following four unconventional but commercially accessible immunomodulatory agents that can be employed in clinical trials to evaluate their effectiveness at alleviating disease symptoms and severity: low-dose oral interferon alpha, microdose DNA, low-dose thimerosal, and phytocannabinoids.

## Abstract (precis)

Severe acute respiratory syndrome coronavirus 2 (SARS coronavirus 2, or SARS-CoV-2) is the cause of the respiratory infection known as COVID-19. From an immunopathological standpoint, coronaviruses such as SARS-CoV-2 induce increased levels of a variety of T-helper 1 (Th1) and inflammatory cytokines and chemokines, including interleukin-1 (IL-1), IL-6, CCL2 protein, and CXCL10 protein. In the absence of proven antiviral agents or an effective vaccine, substances with immunomodulatory activity may be able to inhibit inflammatory and Th1 cytokines and/or yield an anti-inflammatory and/or Th2 immune response to counteract COVID-19 symptoms and severity.

## OPINION/HYPOTHESIS

Severe acute respiratory syndrome coronavirus 2 (SARS coronavirus 2, or SARS-CoV-2) is a recently discovered coronavirus capable of causing the 2019 to 2020 respiratory infection known as COVID-19. Symptoms range from fever and coughing to pneumonia or severe respiratory distress (e.g., shortness of breath). It is related to the coronaviruses responsible for severe acute respiratory syndrome (SARS) from 2002 to 2003 and Middle East respiratory syndrome (MERS), first reported in 2012. Worldwide, over 8,400 people became sick with SARS, of whom over 800 died. For MERS, close to 2,500 cases have been detected, with about 850 related deaths (data from World Health Organization and National Institutes of Health websites). COVID-19 is widespread and, in the midst of this global pandemic, as of late April 2020, there were about three million confirmed infections and over 200,000 deaths reported worldwide (for updates, see the Johns Hopkins University coronavirus COVID-19 dashboard: https://www.arcgis.com/apps/opsdashboard/index.html#/bda7594740fd40299423467b48e9ecf6). To date, there have been no agents proven to be capable of countering the virus, and vaccine candidates are in early clinical testing, with availability to the general public at least a year away.

From an immunological standpoint, coronaviruses cause increases in the levels of T-helper 1 (Th1) cytokine interferon (IFN) gamma, of inflammatory cytokines such as interleukin-1 (IL-1), IL-6, and IL-12, and of related cytokines and chemokines, including IL-8, chemokine (C-C motif) ligand 2 (CCL2 protein, also known as monocyte chemoattractant protein-1, or MCP-1) and C-X-C motif chemokine 10 (CXCL10 protein, also known as interferon gamma-induced protein 10, or IP-10) (1–7). The “cytokine storm” mediated by these inflammatory and Th1 cytokines activates monocytes/macrophages and neutrophils and is responsible for the immunopathological consequences of the infection. It is recognized that hyperinflammatory immune responses can result in increased disease severity and mortality. Therefore, inhibition of the hyperinflammatory response is a definitive drug therapy objective. It has been proposed that certain biological response modifiers, notably, cytokines IL-37 and IL-38, have the potential to inhibit proinflammatory cytokines such as IL-6 and/or to induce an anti-inflammatory immune response or an immunomodulatory response that could counteract COVID-19 patients’ hyperinflammatory responses (8–10). However, the time it might take to develop such cytokine products for the treatment of COVID-19 patients is unknown at this time. There is an urgent need for substances that can potentially counter the effects of SARS-CoV-2 and alleviate the symptoms and severity of COVID-19. In the current situation, every avenue of health care that might be available to decrease morbidity and disease symptoms and severity and promote survival may be worthy of investigation. Accordingly, it is suggested that clinical trials could be conducted on certain substances with immunomodulatory activity from the realm of complementary and alternative medicine. These immunomodulatory agents, while unconventional in nature, offer potential treatment advantages and could augment or possibly be used in place of standard clinical treatments. Furthermore, these potential immunomodulatory agents may be readily available for utilization in clinical trials sanctioned by the U.S. Food and Drug Administration (FDA) or other government drug regulatory agencies. This report discusses four such agents, which were selected based on our prior research, conducted both independently and collaboratively. They are (i) low-dose oral interferon alpha (IFN-alpha), (ii) microdose DNA, (iii) low-dose thimerosal, and (iv) oral or inhalable (by inhaler, not by combustion) phytocannabinoids.

## 

### Low-dose oral IFN-alpha.

IFN-alpha is a cytokine that is a known inducer of antiviral immune responses. There have been commercially available, injectable versions of IFN-alpha (e.g., IFN-alpha-2b [Roferon]), approved by the FDA for use only in cases of chronic hepatitis C and certain forms of cancer. IFN-alpha-2b is dosed at 3 to 9 million international units (IU) and has substantial side effects (see https://www.drugs.com/pro/roferon-a.html). One other noteworthy use of IFN-alpha has been in the treatment of Behcet’s disease, an inflammatory blood vessel disease with a cytokine profile that has been characterized as Th1-like in nature (11). In contrast to formulations such as IFN-alpha-2b, oral (oromucosal) administration of human or bovine IFN-alpha at low doses of 50 to 200 units has been investigated as a potential antiviral strategy for several decades. There have been substantial *in vitro*, *in vivo*, and human and veterinary clinical research studies involving the use of low-dose oral IFN-alpha against infections caused by herpes and influenza viruses, foot and mouth disease virus, and a variety of bovine respiratory viruses (12–14). From a mechanistic standpoint, low concentrations of IFN-alpha can regulate the expression of a variety of cytokine genes, chemokine genes, and related genes involved in antiviral immune responses. In one such study involving peripheral blood mononuclear cells (PBMCs) from calves treated with 50 or 200 units of oral IFN-alpha, the expression levels of 41 of 92 tested autoimmune and inflammatory response-associated genes were significantly upregulated or downregulated (15). Using the Kyoto Encyclopedia of Genes and Genomes (KEGG) online database (https://www.genome.jp/kegg/), 12 of these genes were identified as involved in cytokine-cytokine receptor interactions. What was particularly intriguing was that seven of these genes (CSF1, CXCL12, FAS, IL2RA, IL6R, TNFRSF1A, and TNFSF13B) were downregulated at the 50-U concentration, whereas five of these genes (IFNAR2, IL1A, IL1B, IL-10, and IL10RB) were upregulated at the 200-U concentration. Increased production of cytokine IL-10 (encoded by the IL-10 gene) mediated by IFN-alpha was a key finding in an *in vitro* study of PBMCs derived from Behcet’s disease patients (16). The investigators related the effectiveness of administration of IFN-alpha in treatment of diseases such as Behcet’s disease to changes in Th1 and inflammatory cytokine levels. These data suggest that low-dose oral IFN-alpha can regulate the expression of specific immune response genes and the production of specific cytokines or chemokines that may be relevant to the alleviation of COVID-19 symptoms. While the use of low-dose oral interferon as prophylaxis for influenza in a double-blind, FDA-authorized clinical trial did not prevent acute respiratory illness in treated relative to control individuals, it did reduce symptom severity and was seen as beneficial to a subpopulation of patients (17). Currently, it is marketed as a nutraceutical under the trade name of Paximune. Given the body of existing research and the unmet medical needs of COVID-19 patients, plus a favorable safety profile at these dose levels, it is believed that an FDA-authorized clinical trial of this substance specifically for reducing the symptoms and severity of respiratory symptoms in COVID-19 patients could be conducted in relatively short order.

### Microdose DNA.

Cystic fibrosis (CF) is a genetic disease characterized by abnormal, viscous mucus secretions. The viscosity of these secretions results from the high concentration of exogenous DNA that is released from necrotic neutrophils (18). This observation resulted in the development of the DNA-degrading enzyme, DNase (dornase alfa [Pulmozyme]) as a treatment for CF symptoms (19). Since the presence of excessive neutrophils in the sputum of CF patients suggested an aberrant compensatory immune response, it was hypothesized that the use of exogenous, sublingually administered DNA can be applicable as a neutralization therapy. This hypothesis was the basis for the development of a proprietary formulation of a low concentration of DNA fragments derived from salmon sperm DNA, otherwise referred to as microdose DNA (20, 21). The term “microdose” was applied based on its oral (sublingual) administration in microgram-range doses (0.6 μg per dose, based on a 12 μg/ml DNA concentration and a drop volume of 50 μl). It was hoped that sublingual dosing with microdose DNA would decrease neutrophil necrosis and DNA release into the lungs, thus decreasing sputum viscosity. Microdose DNA was first utilized in evidence-based clinical testing of CF patients. This sublingual therapeutic approach subsequently was extended to patients with other respiratory diseases and otitis media. The specific mechanism(s) of action of microdose DNA has not been elucidated; at least five different hypotheses have been postulated. Among these hypotheses are those positing the generation of beneficial immune responses through increases in levels of anti-inflammatory cytokines and immunomodulatory changes in T-helper 1/T-helper 2 (Th1/Th2) cytokine ratios (21). There is some experimental evidence from *in vivo* studies of dogs with kennel cough indicating that microdose DNA treatment increases levels of the anti-inflammatory cytokine IL-4 (S. W. Mamber, unpublished data). It has also been observed that the DNA fragments might contain oligodeoxynucleotides (ODNs) with the CpG motif (CpG ODNs), which are known to stimulate an immune response to viral infections (22). Under the clinical trial names HP-3 and ML-03, microdose DNA was tested in three separate FDA-approved, placebo-controlled, double-blind phase II clinical trials, one for the treatment of CF, one for chronic bronchitis, and one for chronic obstructive pulmonary disease (COPD). There were only 17 treatment patients and 20 patients on placebo in the CF clinical trial. Although the trials were underpowered to achieve statistical significance, a trend toward improvement was observed for three respiratory parameters. In the chronic bronchitis trial, 25 patients were administered microdose DNA, with 24 patients on placebo. Among other endpoints, there was a statistically significant improvement (*P* = 0.007) in forced expiratory volume (FEF_25–75%_), a measure of small-airway function. Finally, in the COPD clinical trial, there were 23 patients randomized to receive microdose DNA versus 25 on placebo. There was a statistically significant outcome (*P* = 0.019) in a key endpoint, the 6-min walk test (21). All three clinical trials demonstrated the potential of microdose DNA in improving respiratory function in patients with different lung diseases. Moreover, there were no safety issues apparent in these trials. Though it was not developed further as a pharmaceutical agent for economic reasons, the current DNA-based therapeutic is being marketed as a nutraceutical and is being sold commercially as Mucolyxir. The combined evidence-based and clinical trial experiences with various respiratory ailments, plus commercial availability, make microdose DNA a viable candidate to test in clinical trials for treatment of COVID-19 respiratory symptoms.

### Low-dose thimerosal.

Thimerosal (alternatively, thiomersal) is an organomercury compound that is commonly used as a vaccine preservative. With a typical concentration of 0.01%, a 0.5-ml dose of vaccine contains 50 μg of thimerosal. Because of controversy surrounding the presence of thimerosal in vaccines and neurological diseases such as autism, the use of thimerosal in vaccines has been curtailed over the past 20 years (see https://www.fda.gov/vaccines-blood-biologics/safety-availability-biologics/thimerosal-and-vaccines). However, some researchers have been intrigued by the possibility that very small doses of thimerosal (0.2 μg, or 1/250 of the amount present in a typical vaccine dose) can promote an antiviral immune response. In that regard, low-dose thimerosal might be considered to be a hormetic, a substance that is beneficial at low concentrations but inhibitory or toxic at higher concentrations. In terms of background, in 1979 J. B. Miller reported that influenza vaccine could also be used to treat herpesvirus infections (23). In studying the components of the influenza vaccine, it was eventually determined that the antiherpes activity was not related to any influenza virus component of the vaccine. Rather, it was the thimerosal that was responsible (24). Further research indicated that low-dose thimerosal was not acting directly against herpes virus, influenza virus, or other viruses. Instead, low-dose thimerosal might have been signaling an antiviral host response that is immunological in nature. In separate studies, thimerosal has been shown to induce the Th2 immune response and/or inhibit the production of proinflammatory cytokines and chemokines, including IFN-gamma, IL-1 beta, IL-6, IL-12p70, and MCP-1 (25, 26). Furthermore, *in vitro* gene expression profiling experiments with human diploid fibroblast cells indicated that thimerosal at low concentrations (1.6 to 40 ng/ml) can regulate the expression of specific cytokine, chemokine, and related immune response genes capable of mediating host immune responses to viral infections (Mamber, unpublished). Thimerosal can inhibit herpesvirus activity, based on *in vitro* experiments showing viral plaque reduction in treated human keratinocytes, but this is believed to result from innate cellular immune responses rather than from direct antiviral effects (V. Gurel, unpublished results). Low-dose thimerosal is currently not commercially available. However, it has been employed in two FDA-approved, randomized, double-blind, placebo-controlled clinical trials to evaluate its safety and efficacy. The first trial, a phase lla study, evaluated thimerosal for its ability to block progression to lesion in patients with recurrent oral herpes caused by dental trauma, while the follow-up phase llb study evaluated the same indication in patients with herpes caused by exposure to ultraviolet radiation. While the individual clinical trials were underpowered and did not show statistically significant outcomes, the outcome data pooled from the two studies that shared a common endpoint did achieve statistical significance (Beech Tree Labs, unpublished data). There has been little experience in employing low-dose thimerosal against coronaviruses to date. However, the favorable safety profile and the simple formulation and sublingual dosing of low-dose thimerosal make this an interesting candidate for a clinical trial to determine if it can effectively alleviate COVID-19 symptoms and severity. (To further ensure safety, in accordance with thimerosal-containing vaccine recommendations by the FDA, low-dose thimerosal should not be administered to children under 6 years of age.)

### Phytocannabinoids.

Phytocannabinoids derived from Cannabis sativa, such as cannabidiol (CBD) and 9-tetrahydrocannabinol (THC), have been shown to inhibit inflammatory and Th1 cytokines and/or promote anti-inflammatory and Th2 immune responses both *in vitro* and *in vivo* (27–29). As COVID-19 represents a respiratory disease with a dominant Th1 and inflammatory immune response profile, it has been postulated that cannabinoids represent a class of compounds with the potential to alleviate COVID-19 symptoms and severity by helping to decrease inflammation and restore a Th1/Th2 balance in the immune system. THC, for example, has been shown to shift the Th1/Th2 cytokine balance in human T cells to one favoring Th2 cytokines. Of particular interest was the inhibition of IFN-gamma production (27). CBD decreased inflammation in a mouse model of lung injury, with decreased production of proinflammatory cytokines and chemokines, including IL-6 (28). In preliminary studies, an oil extract from Cannabis sativa containing both CBD and THC upregulated Th2 and anti-inflammatory genes such as the IL-4 gene (encoding IL-4) and the PPARG gene (encoding peroxisome proliferator-activated receptor gamma) in human small-airway epithelial cells *in vitro*. There were also certain genes involved in mucus overproduction or hypersecretion that were downregulated. These included the CLCA1 gene (encoding chloride channel accessory 1) and the CMA1 gene (encoding mast cell chymase 1) (29). Preliminary *in vivo* testing in Caribbean vervet monkeys (Chlorocebus aethiops *sabaeus*) indicated that the oil extract improved inspiratory lung functions (J. Osborn, University of Kentucky, unpublished data). More research will be needed to determine which cannabinoid or cannabinoid mixture might be effective in treating COVID-19 symptoms and at what concentrations. The method of drug delivery is also a consideration. The use of combustible products (i.e., smoking) is obviously contraindicated for patients with acute respiratory distress. Oral ingestion would be the logical delivery method. However, an oil-based product may be suitable as the active pharmaceutical ingredient (API) for direct inhalation therapy (e.g., utilization in handheld aerosol inhalers or by nebulized vapor inhalation, which may be augmented with oxygen). API formulation incipient propellants often use natural oil components. Such a formulation would offer a convenient treatment method through delivery to the lungs.

### Comment.

The four substances described here do not have, or are not expected to have, direct antiviral activity against SARS-CoV-2 *in vivo*. (Pending further research, phytocannabinoids may be an exception, which actually would be a positive.) Rather, they appear to be acting as immunomodulatory agents. Modulation of the immune response may be achieved through inhibition of inflammatory cytokines and production of anti-inflammatory cytokines; restoring the Th1/Th2 balance; or otherwise signaling cells to produce therapeutically beneficial cytokines, chemokines, and related proteins. Accordingly, such treatments may have the potential to alleviate the immunopathological symptoms caused by SARS-CoV-2. On the basis of existing *in vivo* and clinical experiences, the optimal time for use of these potential immunomodulatory agents would be at the first signs of disease symptoms, when there would be a better chance of reestablishing immune homeostasis. One further consideration is the potential disease-modifying utility that these immunomodulators might have in patients with preexisting health conditions, including chronic respiratory diseases such as COPD. Such patients may be at the highest risk for severe morbidity and mortality from COVID-19. If formal clinical trials are not feasible, it is suggested that these substances be investigated in an observational manner under principles of informed consent and compassionate use.
